# Associations between maternal and environmental exposures on atopic disease in the offspring of mothers with asthma

**DOI:** 10.1002/iid3.441

**Published:** 2021-06-19

**Authors:** Zarqa Ali, Gregor B.E. Jemec, Charlotte Suppli Ulrik

**Affiliations:** ^1^ Department of Dermatology Bispebjerg Hospital Copenhagen Denmark; ^2^ Department of Dermatology Zealand University Hospital Roskilde Denmark; ^3^ Institute of Clinical Medicine University of Copenhagen Copenhagen Denmark; ^4^ Department of Pulmonary Medicine Hvidovre Hospital Hvidovre Denmark

**Keywords:** lung, skin, tissues

## Abstract

**Background and Objective:**

Available data on the impact of perinatal and environmental factors on atopic diseases in the offspring are sparse and conflicting. We, therefore, investigated the impact of these factors on the risk of atopic diseases in the offspring of women with asthma.

**Methods:**

Pregnant women referred to give birth at Copenhagen University Hospital‐Hvidovre, Denmark, have since 2007 been invited to participate in the Management of Asthma during Pregnancy program. Women with diagnosed asthma, currently prescribed asthma medication, first visit to the respiratory out‐patient clinic within the first 18 weeks of pregnancy that completed an online questionnaire about atopic diseases in their child were included in the current study.

**Results:**

Five hundred and seventy‐one pregnancies were included. Among the off‐spring, 113 children (21%) had doctor‐diagnosed asthma, 178 (31%) atopic dermatitis (AD), and 55 (32%) both AD and doctor‐diagnosed asthma. AD in the offspring was associated with having a dog at home (odds ratio [OR], 2.56; 95% confidence interval [CI], [1.40–4.67], *p* = .002), whereas having a cat at home was associated with a higher risk of asthma in the offspring (OR, 2.16; 95% CI, [1.14–4.11], *p* = .02). The associations remained significant after adjusting for maternal age, smoking status, allergy, treatment with inhaled corticosteroids, forced expiratory volume in 1 s less than 80% predicted, uncontrolled asthma, and history of pre‐pregnancy asthma exacerbations. No association was found between gestational weight gain (GWG) in first trimester and total GWG and atopic disease in the offspring.

**Conclusion:**

Having pets at home is associated with AD and asthma in the offspring of mothers with asthma.

## 1 INTRODUCTION

Atopic dermatitis (AD) is an inflammatory and extremely pruritic skin disease characterized by dry skin.^1^ Loss of function mutations in filaggrin, a skin barrier gene, is an important risk factor for AD.^2^ However, the genetic risk factor does not fully explain the pathology. The etiology is multifactorial with genetics, immune and environmental interaction.^3^ The environmental factor including prenatal exposures, irritants and pruritogens, humidity, air pollutants, and tobacco smoke exposure plays an important role.^3^ Understanding these complex risk factors is crucial in developing a targeted intervention to prevent the atopic disease.

Children with AD have three‐fold increased odds of developing asthma and rhinitis within a five‐year period compared to children without AD.^4^ Approximately 70% of patients with severe AD develop asthma compared with 20%–30% of patients with mild AD^5^ and approximately 8% in the general population.^6^ However, little is known about risk factors for developing concomitant AD, asthma and allergic rhinitis.

The aim of this large‐scale study was to investigate the impact of environmental factors on the risk of asthma, AD, and allergic rhinitis in the offspring of mothers with asthma.

## 2 MATERIALS AND METHODS

### 2.1 Material

In 2007 the management of asthma during pregnancy (MAP) program was initiated. Pregnant women with asthma have since then, through the Department of Gynecology and Obstetrics, Hvidovre Hospital (HVH), been recruited. Around 7000 pregnant women are referred to HVH per year, and they receive information about the program in the standard welcome letter. Women who wanted to participate were given a scheduled appointment at the out‐patient clinic, Department of Respiratory Medicine.

The inclusion criteria for the study are: (1) Asthma diagnosed according to GINA,^7^ (2) currently prescribed asthma therapy rescue treatment with bronchodilator, and (3) First outpatient clinic visit to the respiratory department within the first 18 weeks of pregnancy. Pregnant women with asthma were seen in the respiratory outpatient clinic at recruitment and every four weeks during pregnancy. Information on case history, tobacco exposure, exposure to pets at home, and pre‐pregnancy weight was collected at the first outpatient clinic visit. At each visit spirometry, fractional exhaled nitric oxide (F_E_NO) and maternal body weight were assessed. The MAP program has been described in details elsewhere.^8^, ^9^, ^10^, ^11^, ^12^

All women participating in the MAP program from 2007 to 2015 received an invitation and a reminder by letter from October 2017 to October 2018 to complete an online questionnaire about AD, asthma, and allergic rhinitis in the offspring.

### 2.2 Ethics

The MAP program is approved by the Regional Research Ethics Committee of Denmark (H‐D‐2007‐0051) and conducted in accordance with the Danish legislation.

### 2.3 Definitions and methods

#### 2.3.1 Definitions

Self‐reported pre‐pregnancy weight was used to calculate pre‐pregnancy maternal body mass index (BMI). Underweight was defined as body mass index [BMI] < 18.5, normal weight as 18.5 ≤ BMI < 25 (reference), overweight (25 ≤ BMI < 30, and obese BMI ≥ 30. The difference between self‐reported pre‐pregnancy weight and body weight measured at the first visit was used to calculate gestational weight gain (GWG) in first trimester of pregnancy. The deviation between weight measured at the last visit before the date the women were expecting to give birth and self‐reported pre‐pregnancy weight was total GWG. Asthma‐control was assessed based on asthma symptoms during daytime, awakenings at night due to asthma, use of rescue medication, spirometry, and the level of F_E_NO, and classified as well‐controlled or uncontrolled.^8^, ^10^

Asthma and AD were defined as if a doctor had ever said that their child has asthma or AD. AD are defined according to the U.K. Working Party definition,^13^ as itchy skin plus three or more of the following features: (1) Onset before age 2, (2) history of flexural involvement, (3) A history of asthma and/or allergic rhinitis, (4) A history of generally dry skin in the last year, and (5) Visible flexural dermatitis.

The status of maternal allergy was constructed from previous testing for allergy and patient symptoms, and defined as perennial (i.e., allergy for animals, fungi and dust mite) or seasonal (i.e., allergy for airborne substances [such as pollens]).

SAS Enterprise Guide 7.1 (SAS institute) was used for the analyses. Two‐tailed Student *t* test was used for continuous variables and *χ* ^2^ test for the binary variables. Following confounders were used for the adjusted association: maternal age, smoking status (never smoker vs. ever smoker), treatment with inhaled corticosteroids, forced expiratory volume in 1 s (FEV_1_) < 80% predicted, pre‐pregnancy BMI, pre‐pregnancy asthma exacerbations, uncontrolled asthma, and allergy.

Results are given as odds ratios (OR) with 95% confidence intervals (CI). A *p* < .05 was considered statistically significant.

## 3 RESULTS

### 3.1 Background data

A total of 1208 women (1283 pregnancies) with asthma were invited to complete the questionnaire about atopic diseases in the offspring, that is, AD, hay fever and asthma. Thirteen were excluded due to missing address in Denmark or moving abroad, and 16 were excluded due to incorrect birth date or error in the unique identity number (MAP number) given in the completed questionnaire. A total of 571 pregnancies (46%) were included in the analysis. Baseline characteristics of women with asthma completing the questionnaire and enrolled children are provided in Tables 1 and 2, respectively.

**Table 1:** Baseline characteristics of 571 women with asthma enrolled in the management of asthma during pregnancy (MAP) program

**Characteristics**	**Women with asthma**
	**(*n* = 571)**
Age (years)	31 ± 5 (17‐44)
Atopy in women with asthma	117 (21)
Singleton pregnancies	554 (97)
Pre‐pregnancy self‐reported data	
Weight (Kg)	61.1 ± 12.8 (39‐131)
BMI (Kg/m^2^)	23.9 ± 4.2 (15‐42)
Underweight (BMI < 18.5)	28 (5)
Normal weight (18.5≤BMI < 25)	372 (65)
Overweight (25 ≤ BMI < 30)	112 (20)
Obese (BMI ≥ 30)	59 (10)
Smoking status	
Never smoker	402 (70)
Ever smoker	131 (23)
Pack‐years^a^	7.9 ± 6.1
Current smoker	38 (7)
Pack‐years^a^	11.9 ± 7.8
Lung function	
FEV_1_ < 80% predicted	114 (20)
FEV_1_ ≥ 80% predicted	457(80)
Treatment for asthma	
Rescue bronchodilator only	192 (34)
Prescribed ICS treatment	379 (66)
Daily dose^b^	
Low	83 (14)
Medium	199 (35)
High	97 (17)
Add‐on long‐acting beta‐2 agonist	119 (21)
Add‐on leukotriene receptor antagonist	5 (1)

*Note*: Data are given as mean ± *SD* with range in brackets or numbers with percentages in brackets.

Abbreviations: BMI, body mass index; FEV_1_, forced expiratory volume in 1 s; ICS, inhaled corticoid treatment.

^a^ Pack‐year = (number of cigarettes smoked per day/20) × number of years smoked.

^b^ Beclometasone equivalent doses.

**Table 2:** Baseline characteristics of children of 571 women with asthma enrolled in the MAP program

**Characteristics**	**Offspring**
Gender of offspring (male)	278 (49)
Doctor‐diagnosed asthma	113 (21)
Asthma‐like symptoms	
Ever wheeze	273 (50)
Wheeze^a^	154 (28)
Wheeze at night^a^	131 (23)
Speech‐limiting wheeze^a^	26 (5)
Exercise‐induced wheeze^a^	54 (10)
Dry cough^a^	143 (26)
Doctor‐diagnosed atopic dermatitis (AD)	173 (30)
AD according to UK criteria	178 (31)
Topical prescription	186 (33)
Itchy rash before the age of 2	166 (31)
Itchy rash^a^	215 (38)
Dry skin (past week)	179 (31)
Scaly skin (past week)	74 (12)
Itchy rash (past week)	133 (23)
Sleep disturbance due to itching (past week)	36 (6)
Skin cracks (past week)	59 (10)
Skin bleeding (past week)	27 (4)
Skin weeping (past week)	15 (2)
Allergic rhinitis	72 (13)
Running nose without cold^a^	111 (21)
Itchy eyes^a^	69 13)

*Note*: Data are given as mean ± *SD* with range in brackets or numbers with percentages in brackets.

Abbreviation: MAP, management of asthma during pregnancy.

^a^ Past 12 months

Women responding to the questionnaire were more often first‐time mothers (70% vs. 67%, *p* = .03), and more often never‐smokers (76% vs. 73%, *p* = .02) compared to non‐responders. No difference was found between responders and non‐responders with regard to pre‐pregnancy weight, weight gain in first trimester, total gestational weight gain, FEV_1_/forced vital capacity (FVC)‐ratio, F_E_NO, or year of enrollment into the MAP cohort. The mean age of the included children were 6.1 years (*SD*, 2.13; range 2–12 years).

### 3.2 Doctor‐diagnosed asthma and asthma‐like symptoms in the offspring

A total of 113 (21%) of the children had doctor‐diagnosed asthma. Having a cat at home was a significant risk factor for asthma in the offspring (OR, 2.16; 95% CI, [1.14–4.11]; *p* = .02) (Table 3). The associations remained significant after adjusting for confounders (OR_adjusted_, 2.36; 95% CI, [1.21–4.62]; *p* = .01). Further, ever wheeze (OR 2.34, 95% CI (1.02‐5.35), *p* = .04) and exercise‐induced wheeze (OR, 2.79; 95% CI, [1.05–7.42], *p* = .04) were associated with pre‐pregnancy underweight and remained significant after adjusting for confounders (ever wheeze OR_adjusted_ 2.56; 95% CI, (1.09–6.04); *p* = .03; exercise‐induced wheeze OR_adjusted_, 3.19; 95% CI, (1.13–8.95); *p* = .02).

**Table 3:** The associations between risk factors and atopic disease, that is, asthma, atopic dermatitis, and allergic rhinitis, in the offspring of mothers with asthma enrolled in the MAP program

	**Doctor‐diagnosed asthma**	**Atopic dermatitis**	**Allergic rhinitis**
	**OR (95% CI)**	**OR (95% CI)**	**OR (95% CI)**
Maternal risk factors			
Smoking	0.99 (0.63–1.57)	1.32 (0.91–1.92)	2.03 (1.22–3.39)^a^
Age >35 years	0.85 (0.47–1.53)	1.40 (0.88–2.22)	0.73 (0.35–1.52)
Primiparity	1.06 (0.68–1.66)	1.08 (0.74–1.57)	0.96 (0.57–1.64)
Maternal asthma			
FEV_1_ pred<80%	0.76 (0.44–1.31)	0.77 (0.49–1.20)	0.60 (0.29–1.21)
FeNO >50	0.71 (0.29–1.74)	0.8 (0.41–1.68)	0.53 (0.16–1.80)
Uncontrolled asthma	0.99 (0.63–1.58)	1.41 (0.96–2.05)	1.48 (0.88–2.48)
Maternal allergy			
Perennial allergy	1.02 (0.67–1.55)	1.18 (0.83–1.67)	0.79 (0.49–1.31)
Seasonal allergy	1.03 (0.67–1.57)	1.20 (0.84–1.71)	1.05 (0.63–1.74)
Pre‐pregnancy BMI			
<18.5	1.82 (0.76–4.33)	2.31 (1.07–5.00)^a^	1.42 (0.51–3.94)
18.5 ≤ BMI < 25	Reference	Reference	Reference
25 ≤ BMI < 30	1.23 (0.73–2.08)	0.99 (0.63–1.55)	0.72 (0.36–1.45)
30 ≤ BMI	1.34 (0.69–2.59)	1.11 (0.62–1.96)	0.98 (0.44–2.20)
GWG in first trimester, kg			
<3	1.09 (0.50–2.39)	0.69 (0.37–1.29)	1.55 (0.53–4.59)
3–5	Reference	Reference	Reference
≥5	0.86 (0.35–2.09)	0.80 (0.40–1.62)	1.54 (0.47–5.00)
Total GWG, kg			
<10	0.92 (0.57–1.50)	0.99 (0.65–1.49)	1.02 (0.56–1.87)
10–15	Reference	Reference	Reference
15‐20	1.01 (0.51–2.00)	1.29 (0.73–0.2.28)	1.34 (0.61–2.97)
≥20	1.14 (0.45–2.89)	1.26 (0.60–0.2.66)	1.11 (0.35–4.56)
Pets			
Dog at home	0.78 (0.351.71)	2.56 (1.40–4.67)^a^	0.80 (0.31–2.10)
Cat at home	2.16 (1.14–4.11)^a^	1.27 (0.69–2.33)	1.62 (0.74–3.51)

*Note*: Results are reported as OR with 95% CI. Atopic dermatitis is defined according to UK criteria.

Abbreviations: BMI, body mass index; CI, confidence interval; GWG, gestational weight gain; MAP, management of asthma during pregnancy; OR, odds ratio.

^a^ Significant.

### 3.3 Atopic dermatitis in the offspring

A total of 173 (30%) children of the enrolled mothers with asthma had doctor‐diagnosed AD and 197 (35%) had AD according to the UK criteria. Only 89 (45%) children fulfilling the UK criteria had doctor‐diagnosed AD (Table 1).

AD according to the UK criteria was associated with having a dog at home (OR, 2.56; 95% CI, [1.40–4.67]; *p* = .002) and pre‐pregnancy underweight (OR, 2.31; 95% CI, [1.07–5.00), *p* = .04) (Table 3). The associations for having dog at home (OR_adjusted_, 2.35; 95% CI, [1.27–4.35]; *p* = .007) and pre‐pregnancy underweight (OR_adjusted_, 2.32; 95% CI, [1.03–5.19]; *p* = .04) remained significant after adjusting for confounders. Furthermore, no association was found between GWG in first trimester and total GWG and AD in mothers with asthma with pre‐pregnancy underweight.

### 3.4 Allergic rhinitis

Cigarette smoking during pregnancy remained significantly associated with allergic rhinitis in the offspring even when the association was adjusted for confounders (OR_adjusted_, 2.06; 95% CI, [1.21–3.51], *p* = .01).

### 3.5 Concomitant atopic dermatitis, asthma, and allergic rhinitis

A total of 57 children (29%) with AD also had doctor‐diagnosed asthma, and 26 (13%) had AD, asthma plus allergic rhinitis. No associations were found significant for AD plus asthma, and AD, asthma plus allergic rhinitis.

All the analyses were repeated when women contributing with more than one pregnancy and twin pregnancies were excluded; however, it did not change the overall results.

## 4 DISCUSSION

The present study of over 500 pregnancies showed that having pets at home is associated with AD and asthma in genetic predisposed children (i.e., having a mother with asthma). Further, pre‐pregnancy underweight is a risk factor for AD and ever wheeze in the offspring of mothers with asthma.

### 4.1 Prevalence of AD and asthma

The prevalence of AD in Danish children is 13%.^14^ The prevalence can be as high as 30%–50% if at least one of the parent have a history with atopy.^15^ We found a prevalence of 30%, which is expected as our cohort consist of mothers with asthma. The risk of asthma in children with AD is elevated and the prevalence of asthma in children with AD is reported around 30%.^16^ In present study 32% of children with AD according to UK criteria had doctor‐diagnosed asthma.

### 4.2 Pets and AD

Cat and dog allergens are commonly found in public places and around 90% of homes in the United States have measurable levels of these allergens whether the home has ever had a furred pet living in it or not.^17^ The studies exploring the association between pets and atopic diseases have been contradictory so far.^18^, ^19^ Though, systematic reviews have reported reductions in the risk of atopic diseases among children with close contact with animals (farm animals or indoor pets) during infancy.^20^, ^21^, ^22^ However, regarding AD some studies have found a protective association of pets,^23^ and others studies with adjustment for avoidance behavior have not found this association.^24^ Bisgaard et al.^25^ have in two different cohorts shown that cat, but not dog, exposure in the home is a statistically significant risk factor for AD in children with filaggrin mutation.

On the other hand exposure to cat have been associated with increased risk of asthma,^26^ and increased bronchial responsiveness.^27^ In line with this we also found increased risk of doctor diagnosed asthma in children having cats at home. On the contrary dog exposure during the first year of life have been associated with a decreased risk of asthma in school‐aged children.^28^

### 4.3 Gestational weight gain and risk for AD and asthma in the offspring

The prevalence of obesity ^29^ and excessive GWG during pregnancy ^30^ have increased substantially over the last few decades. It has been suggested, that maternal obesity may have an impact on maternal asthma and contribute to asthma in the offspring.^31^, ^32^ Obesity is considered to be a condition of chronic low‐grade systemic inflammation,^33^ and the impact of both maternal weight and GWG are probably facilitated through proinflammatory part ways and elevated cytokine levels.^32^, ^34^, ^35^ The inflammation triggered by GWG may have an impact on atopic diseases in the offspring through the same proinflammatory mechanisms that affects maternal asthma. High GWG have been linked to asthma in the offspring in several studies.^36^, ^37^, ^38^, ^39^ However, these studies did not enroll atopic or more specific mothers with asthma, where a synergistic or additive effect of the risk factors may be hypothesized. In the present study there was no association between GWG in first trimester or total GWG and asthma in the offspring of mothers with asthma. The incidence of asthma is strongly related to family history ^40^ and children with a parent with asthma are almost twice as likely to develop asthma compared to those without a parent with asthma.^41^ It is likely that the genetic component is a strong predictor for childhood asthma that the inflammation, caused by the weight gain, in the uterus during pregnancy does not play an important role in asthmatic. Previous studies have also suggested that the elevated risk of asthma in the offspring is caused by GWG or maternal obesity is particularly significant for mothers without asthma history.^37^

There is inconsistency in results regarding AD. Some studies find a positive association between increasing GWG and AD in the child^42^, ^43^ and others do not find this association.^36^

Known risk factor for AD are filaggrin gene mutations or ceramide deficiency ^44^ and a positive family history of atopic disease in either parent, the last one have shown to confer a greater risk of developing AD.^45^ It is likely that maternal family history is a strong determinant of AD that exceed the possible effect of GWG if any.

### 4.4 Pre‐pregnancy underweight

Pre‐pregnancy underweight was associated with both ever wheeze and AD in the offspring. However, it should be emphasized that there was no statically significant difference in excessive weight gain during first trimester or during the entire pregnancy between women with pre‐pregnancy underweight and women that were not underweight before pregnancy.

Underweight may be a consequence of chronic disease or an inappropriate diet that might result in suboptimal fetus nutrition. Growth restriction intrauterine is a risk factor for reduced lung function and respiratory diseases in the child.^46^ Further, pre‐pregnancy underweight increases the risk of small for gestational age and low birth weight in the infant^47^ and low birth weight significantly increases the risk of childhood asthma.^48^ Moreover, lower first trimester crown‐rump length is associated with greater risk of AD.^49^

## 5 STRENGTH AND LIMITATION

All women were seen by the same pulmonologist (CSU) at first visit and this makes the diagnosis of maternal asthma very reliable as it was made on the basis of a combination of previous findings, current symptoms and objective assessment at the visit to the respiratory out‐patient clinic. Maternal weight was measured at all visits throughout pregnancy. However, pre‐pregnancy weight was reported by the women. Nevertheless, self‐reported pre‐pregnancy weight are validated and correlates well with actual weight.^50^

Doctor‐diagnosed asthma in the offspring was generated from the question: “Has a doctor ever said that your child had asthma?.” The specificity of the question of “doctor‐diagnosed asthma” has been found to be 99%.^51^

It is a strength that the analysis was adjusted for tobacco exposure, as this may not only influence asthma but AD as well.^52^ There was however a discrepancy in doctor‐diagnosed AD and AD defined according to UK criteria. The cohort consisted of atopic mothers. It is likely, that either these women themselves or a family member have AD. The women have probably used topical treatments without prescription to treat mild to moderate AD without consulting a doctor. This may explain why as little as 45% of the children fulfilling the UK criteria had doctor‐diagnosed AD.

The response rate of the present questionnaire was 46%. A response rate of 50%–60% or greater is optimal because nonresponse bias is thought to be minimal.^53^ However, studies with a low response rates, as low as 20%, are often only marginally less accurate than those with much higher described response rates.^54^ There is no difference in the responders and non‐responders in the present study according to pre‐pregnancy weight, weight gain in first trimester, total GWG, FEV_1_/FVC‐ratio, F_E_NO, or year of enrollment into the MAP cohort. The women responding to the questionnaire is therefore considered representative of the entire MAP cohort. There is a risk of bias in population studies associated with lifestyle. We have shown that at our department there is a higher proportion of nulliparous women accepting an invitation to participate in lifestyle interventional trials during pregnancy.^55^ We also found an overweight of woman who has never given birth (nulliparous) in this study. Further, regarding age and marital status, women enrolled in the MAP cohort and women from the background population are comparable.^8^

Finally, one important limitation needs to be addressed. Children born early in the MAP program were more than 5 years older than the youngest children born in the MAP program. The older children have had more time to be diagnosed with asthma compared to the younger children. Further, some children may be so young that they could not get the asthma diagnosis at the time the questionnaire was completed.

In the present study we found a positive association between having pets at home and developing AD and asthma. Furthermore, there was a positive association between pre‐pregnancy underweight and AD and ever wheeze in the offspring of mothers with asthma. Understanding these complex risk factors is crucial when clinicians are giving advice to prevent atopic diseases in genetic predisposed children. Future studies should investigate if removing pets from home before the birth and improvement of the maternal nutritional status before pregnancy can decrease the likelihood of AD and wheeze in the offspring.

## CONFLICT OF INTERESTS

The authors declare that there are no conflict of interests.

## Data Availability

All data is available upon request.
